# A Bi-GRU-attention neural network to identify motor units from high-density surface electromyographic signals in real time

**DOI:** 10.3389/fnins.2024.1306054

**Published:** 2024-03-13

**Authors:** Chuang Lin, Chen Chen, Ziwei Cui, Xiujuan Zhu

**Affiliations:** Information Science and Technology College, Dalian Maritime University, Dalian, China

**Keywords:** real-time decomposition, motor units, blind source separation, gate recurrent unit network, attention

## Abstract

To utilize surface electromyographics (sEMG) for control purposes, it is necessary to perform real-time estimation of the neural drive to the muscles, which involves real-time decomposition of the EMG signals. In this paper, we propose a Bidirectional Gate Recurrent Unit (Bi-GRU) network with attention to perform online decomposition of high-density sEMG signals. The model can give different levels of attention to different parts of the sEMG signal according to their importance using the attention mechanism. The output of gradient convolutional kernel compensation (gCKC) algorithm was used as the training label, and simulated and experimental sEMG data were divided into windows with 120 sample points for model training, the sampling rate of sEMG signal is 2048 Hz. We test different attention mechanisms and find out the ones that could bring the highest F1-score of the model. The simulated sEMG signal is synthesized from Fuglevand method (J. Neurophysiol., 1993). For the decomposition of 10 Motor Units (MUs), the network trained on simulated data achieved an average F1-score of 0.974 (range from 0.96 to 0.98), and the network trained on experimental data achieved an average F1-score of 0.876 (range from 0.82 to 0.97). The average decomposition time for each window was 28 ms (range from 25.6  ms to 30.5 ms), which falls within the lower bound of the human electromechanical delay. The experimental results show the feasibility of using Bi-GRU-Attention network for the real-time decomposition of Motor Units. Compared to the gCKC algorithm, which is considered the gold standard in the medical field, this model sacrifices a small amount of accuracy but significantly improves computational speed by eliminating the need for calculating the cross-correlation matrix and performing iterative computations.

## 1 Introduction

The electrical measures of signals in the human body that carry information regarding motor intention are predominantly comprised of Electroencephalogram (EEG) signals, electromyographic signals (EMG), and peripheral nerve signals. Among these, the collection of electromyographic signals is simpler and their processing is more convenient. Crucially, electromyographic signals can be collected even before actual force of exercise takes place (Hashemi et al., 2015). By using EMG to infer central control strategies, it is possible to comprehend the physiological processes that lead to muscle activity for generating strength, performing exercises, and facilitating various functions (Farina et al., 2004). EMG signals serve as indicators of muscle activity initiation, allowing us to obtain the temporal relationship of one or multiple muscles during task execution. Furthermore, EMG signals can provide information on force contribution of the individual muscle or muscle group. Due to these characteristics of EMG, our ways of interacting with the world may become more enriched, and can also be useful for medical and health monitoring and diagnosis (De Luca, 1997).

The human central nervous system controls muscle strength by modulating the activity of motor units (MU), which are composed of motor neurons in the ventral horn of the spinal cord, along with the axons and muscle fibers innervated by them. According to the principles of EMG signal formation, the decomposition refers to extracting two key components from EMG signals: motor unit action potentials (MUAP) and the pulse trains of MU. Blind source separation can be employed in the medical field for health monitoring and diagnosis to infer the central control strategies of the human body (Negro et al., 2016), since pulse trains obtained from decomposing EMG signals can extract human body data, which means pulse trains can be quantified and used for experimental analysis of motor neurons. S. P. Strong’s teams have successfully implemented the quantification of information from Pulse Trains using bits as the unit, thereby demonstrating that Pulse Trains carry a significant amount of information (Strong et al., 1998). The movement information transmitted by EMG signals can be extracted through their decomposition. The evaluation of MUAP can assist in clinical disease diagnosis based on morphological and biological characteristics, while studying MU pulse sequences can provide reference for diagnosing central nervous system dysfunction (Heckman and Enoka, 2004). Additionally, based on pulse trains, researchers can analyze the behavioral relationships between different MUs during muscle contraction, providing more effective research on the working characteristics both within and between muscles. When muscles contract, analyzing the overall signal information to determine the degree of contraction during exercise can provide a deeper understanding of the normal function of the neuromuscular system (Heckman and Enoka, 2004). Recently, there have been applications of utilizing Pulse Trains for electromyographic control, achieving the decoding of both discrete and continuous human movements. Chen’s team successfully employed Pulse Trains to recognize discrete gestures and achieve continuous prediction of wrist torque (Chen et al., 2020, 2023).

To utilize EMG for control purposes, it is necessary to perform real-time estimation of the neural drive to the muscles, which involves real-time decomposition of the EMG signals. For accurate motion intention prediction, the delay between samples must fall within a specific time range of 225 ± 50 ms (Del Vecchio et al., 2018). Several traditional blind source separation algorithms, including mature algorithms such as gradient convolution kernel compensation (gCKC) (Holobar and Zazula, 2007), compute innervation pulse trains (IPTs) using separation vectors to generate Pulse Trains. Online decomposition becomes feasible after calculating the separation vector that corresponds to MU. The gCKC algorithm achieves high accuracy in MU decomposition through iterative calculations. In the field of EMG decomposition, fast independent component analysis (fastICA) (Hyvarinen, 1999) has also made notable contributions due to its ability to converge quickly and not require the setting of step parameters. However, the iterative algorithm operation renders it unsuitable for meeting the demands of online decomposition.

Recently, neural network-based blind separation methods have emerged. For example, the IPT prediction, which is based on the gate recurrent unit network (GRU), exhibits higher robustness compared to the gCKC algorithm under low signal-to-noise ratio settings (Clarke et al., 2020). Furthermore, with a processing time of 67 ms per second for sEMG, the IPT prediction demonstrates great potential for online signal decomposition. Furthermore, the online prediction of Pulse Trains using deep convolutional neural networks (DCNN) (Wen et al., 2021) requires approximately 40 ms to predict every 120 sampling points when the EMG sampling rate is 2048 Hz, which is lower than the physical electrical delay range. However, lower delay is necessary to achieve synchronization between devices and users (Zhang et al., 2017).

In this paper, we propose an online decomposition method using a Bidirectional GRU, which achieves real-time multiple MU decomposition while meeting minimum delay requirements. We begin by using the gCKC algorithm to decompose EMG to obtain pulse trains, and then combine them with EMG signal segments as training data. Secondly, we use recurrent neural network (RNN) and some of its variants, attention mechanisms, data processing techniques, and different signal-to-noise ratios (SNRs) of EMGs to build the network and train our blind source separation models. Finally, we analyzed the model’s accuracy performance (i.e., precision, F1-score) and processing time (time required to decompose each sample) under these conditions.

## 2 Materials and methods

### 2.1 Research background and algorithmic foundations

#### 2.1.1 Gradient convolution kernel compensation

The gCKC algorithm estimates the pulse information of MUs from EMG without calculating mixed matrices. An isometric contraction of a muscle is a contraction process in which the tension changes while the length of the muscle remains constant. HD-EMG can be modeled as a linear, time-invariant, convolutional MI-MO system. Assuming there are M EMG signal acquisition channels available, the EMG signal for the i-th channel can be represented as (Garcia et al., 2005):


$$x_{i}\left(n\right)=\overset{N}{\underset{j=1}{\sum }}\overset{L-1}{\underset{l=0}{\sum }}h_{ij}\left(l\right)s_{j}\left(n-l\right),\;i=1,\ldots ,N$$

where $h_{ij}$ is the MUAP of the j-th MU in the i-th channel, and has a length of L. $s_{j}$ is the pulse time series of the j-th MU:


$$s_{j}\left(n\right)=\overset{\infty }{\underset{k=-\infty }{\sum }}\delta \left(n-\psi_{j}\left(k\right)\right),j=1,\ldots ,N$$

where $\delta \left(\text{.}\right)$ is the Dirac impulse, and $\psi_{j}\left(k\right)$ is the occurrence of the k-th MUAP of the j-th MU. During the experimental EMG signal acquisition process, noise is inevitably incorporated into the collected signal. Hence, the EMG signal can be represented as:


$$y_{i}\left(n\right)=x_{i}\left(n\right)+\omega_{i}\left(n\right),i=1,\ldots ,M$$

where $y_{i}\left(n\right)$ is the EMG signal recorded during the acquisition process, and $\omega_{i}\left(n\right)$ is considered to be the Gaussian white noise with a zero-mean value. In order to enhance the numerical conditions of the above model and improve the signal decomposition effect, delay expansion is necessary for each acquisition channel:


$$\begin{array}{l} \bar{y}\left(n\right)=[y_{1}\left(n\right),y_{1}\left(n-1\right),\ldots ,y_{1}\left(n-K+1\right),\ldots ,\\ {\;y_{M}\left(n\right),\ldots ,y_{M}\left(n-K+1\right)]}^{T} \end{array}$$

After sorting out the above formula, we can get:


$$\bar{y}\left(n\right)=H\bar{s}\left(n\right)+\bar{\omega }\left(n\right)$$

where h is a mixed matrix, which can be represented as:


$$\begin{array}{l} H=\\ \;\left[\begin{array}{cccc} h_{11}\left(0\right)\ldots h_{11}\left(L-1\right) & h_{12}\left(0\right)\ldots h_{12}\left(L-1\right) & \ldots & h_{1N}\left(0\right)\ldots h_{1N}\left(L-1\right)\\ h_{21}\left(0\right)\ldots h_{21}\left(L-1\right) & h_{22}\left(0\right)\ldots h_{22}\left(L-1\right) & \ldots & h_{2N}\left(0\right)\ldots h_{2N}\left(L-1\right)\\ \vdots & \vdots & \ddots & \vdots \\ h_{M1}\left(0\right)\ldots h_{M1}\left(L-1\right) & h_{M2}\left(0\right)\ldots h_{M2}\left(L-1\right) & \ldots & h_{MN}\left(0\right)\ldots h_{MN}\left(L-1\right) \end{array}\;\right] \end{array}$$

where $h_{ij}$ is the MUAP of the j-th MU in the i-th channel, which has a length of L.

According to the research of Holobar and Zazula (2007), the pulse trains for the j-th mu can be directly estimated from the extended EMG signal:


$${\hat{s}}_{j}\left(n\right)=c_{{\bar{s}}_{j},\bar{y}}^{T}C_{\bar{y}\bar{y}}^{-1}\bar{y}\left(n\right)$$

where $c_{{\bar{s}}_{j},\bar{y}}=E\left({\bar{s}}_{j}\left(n\right)\bar{y}\left(n\right)\right)$ is the cross-correlation signal between the extended pulse trains and the EMG signal, and $C_{\bar{y}\bar{y}}=E\left(\bar{y}\left(n\right){\bar{y}}^{T}\left(n\right)\right)$ is the correlation matrix of the extended EMG signal. $E\left(\text{.}\right)$ is mathematical expectation.

After undergoing the aforementioned procedure, the EMG signal can be decomposed into pulse sequences of motor units. The formula indicates that only the cross-correlation vector and cross-correlation matrix of the extended signal need to be calculated, without the need to calculate the convolution matrix. By using the gradient descent method, the pulse train of MUs can be estimated directly from the EMG signal. This algorithm is called gradient Convolution Kernel Compensation (gCKC) algorithm.

#### 2.1.2 Gate recurrent unit network

**Figure 1:**
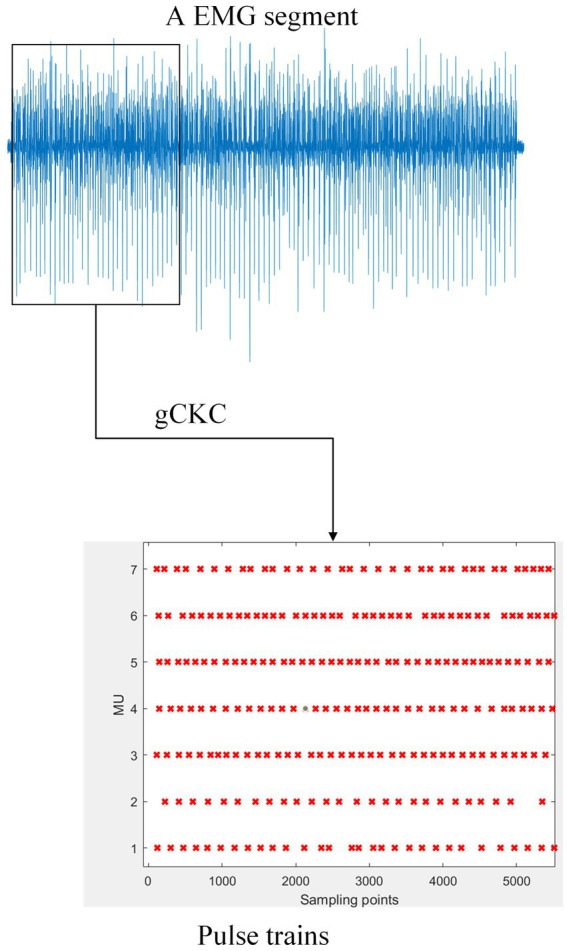
The gCKC algorithm decomposes the mathematically represented EMG signal into multiple MUs’ pulse trains. For example, the schematic of one channel of EMG signal on the blue line in the figure demonstrates this. The gCKC operates on multiple channels of EMG signals, each with a sampling rate of 2048 Hz, and decomposes them into seven MU’s pulse trains. Pulse trains shows that the MU has a pulse at this sampling point with a red “x”.

For RNN’s variants, the Gate Recurrent Unit Network is a notable gating mechanism that has shown rapid progress (Chung et al., 2014). Compared with traditional RNN, GRU can solve issues related to gradient and long-term memory. The GRUis a popular solution in the field of natural language processing (NLP) due to its ability to connect data both backward and forward. The GRU network finds applications in processing continuous time signals, including the EMG signal which is a continuous time signal collected in both time and space. The GRU addresses certain limitations of the Long Short-Term Memory network (LSTM) by integrating the “forgetting gate” and “output gate” into an “updating gate,” resulting in a reduction in training parameters. This leads to significant savings in training time and data requirements without significantly impacting the model’s predictive ability, as reported in the findings (Graves, 2012). Given its computational efficiency and time-saving advantages, GRU continues to be a subject of sustained interest among researchers (Figure 1).

**Figure 2:**
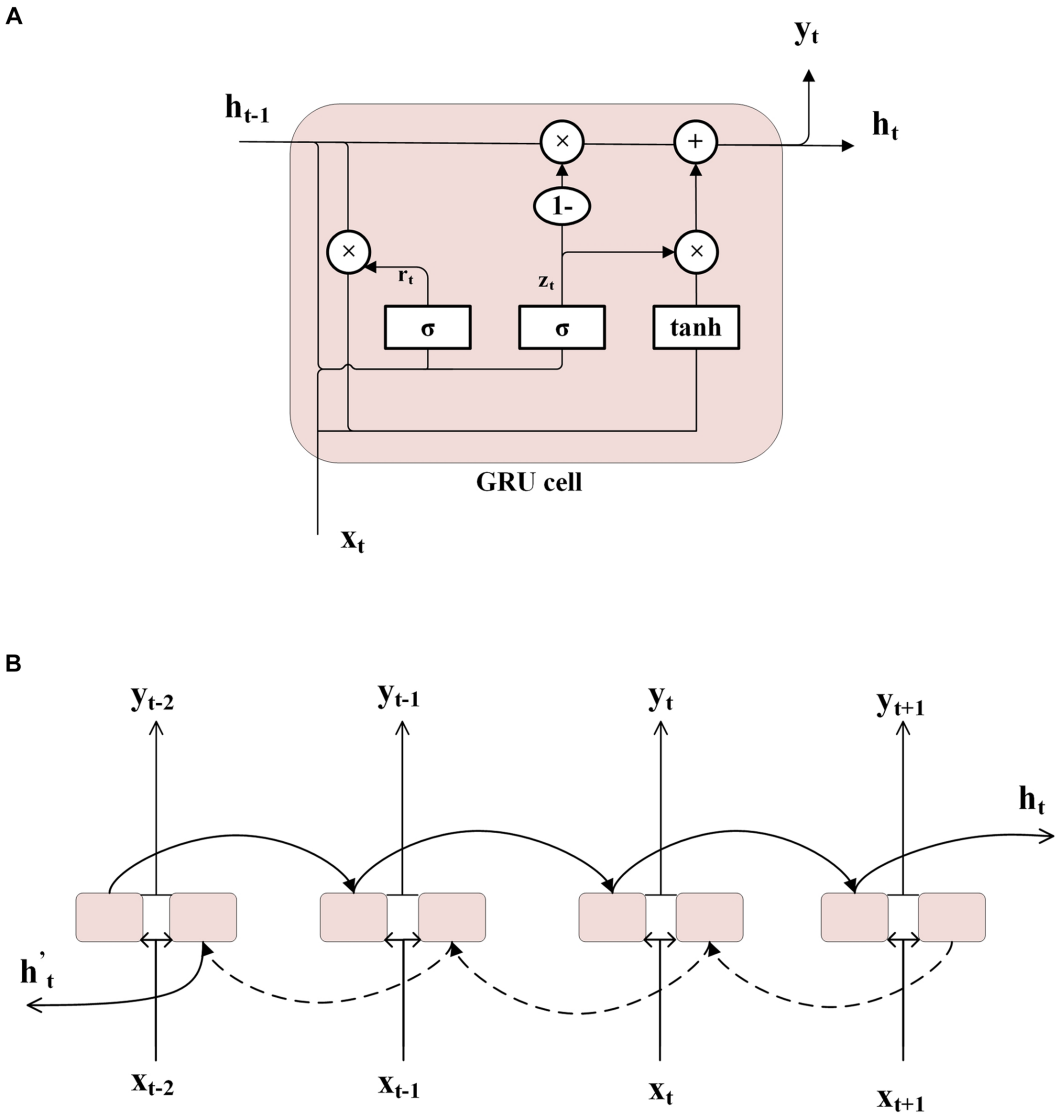
In **(A)**, the internal structure of a GRU unit is shown. **(B)** Illustrates part of the structure of a bi-directional GRU network, where two ordinary GRU networks are used to process the input data in forward and reverse directions, and then integrate the outputs from both.

For one gating unit of GRU (Figure 2A):


$$z_{t}=\sigma \left(W_{z}x_{t}+U_{z}h_{t-1}+b_{z}\right)$$

$$r_{t}=\sigma \left(W_{r}x_{t}+U_{r}h_{t-1}+b_{r}\right)$$

$${\hat{h}}_{t}=\tanh\left(W_{h}x_{t}+U_{h}\left(r_{t}.h_{t-1}\right)+b_{h}\right)$$

$$h_{t}=\left(1-z_{t}\right).h_{t-1}+z_{t}.{\hat{h}}_{t}$$

where the input vector $x_{t}$ and output vector $h_{t}$ are defined, as well as the update gate vector $z_{t}$ and reset gate vector $r_{t}$ W, U and b represent parameter matrices and vectors that require training. The update gate $z_{t}$ updates the memory of the model by combining the output of the previous unit $h_{t-1}$ with the input of the current unit $x_{t}$ and controlling their weights. The reset gate $r_{t}$ determines the combination of the input of the current unit $x_{t}$ with the previous memory $h_{t-1}$ by controlling the weight. Finally, the output vector of the current cell $h_{t}$ is calculated by synthesizing the memory passed from the update gate $z_{t}$ and reset gate $r_{t}$ and the current input vector $x_{t}$ . This calculation enables the GRU to retain information from previous signals in the backward time.

To capture contextual information of the current unit, a Bidirectional Gate Recurrent Unit network (Bi-GRU) is utilized (Figure 2B). This network consists of two parallel GRUs that operate in both forward and backward directions along the time dimension. Each network processes the signal sequence independently, generating outputs that are then concatenated together. Consequently, the output of the Bi-GRU at each time step encompasses relevant contextual information pertaining to the current unit.

#### 2.1.3 External attention

The attention mechanism was first introduced in 2014 (Bahdanau et al., 2014). Initially applied in natural language processing, similar to the GRU model, it has since undergone various developments and adaptations to cater to different fields. Typically, attention is integrated within the encoder-decoder framework, allowing for enhanced performance and effectiveness. When incorporated into the framework, this approach significantly improves the prediction model’s capability to capture relevant local information. Through our experiments with various attention mechanisms in the GRU model, we have observed an enhanced sensitivity of the model toward the EMG pulse component.

**Figure 3:**
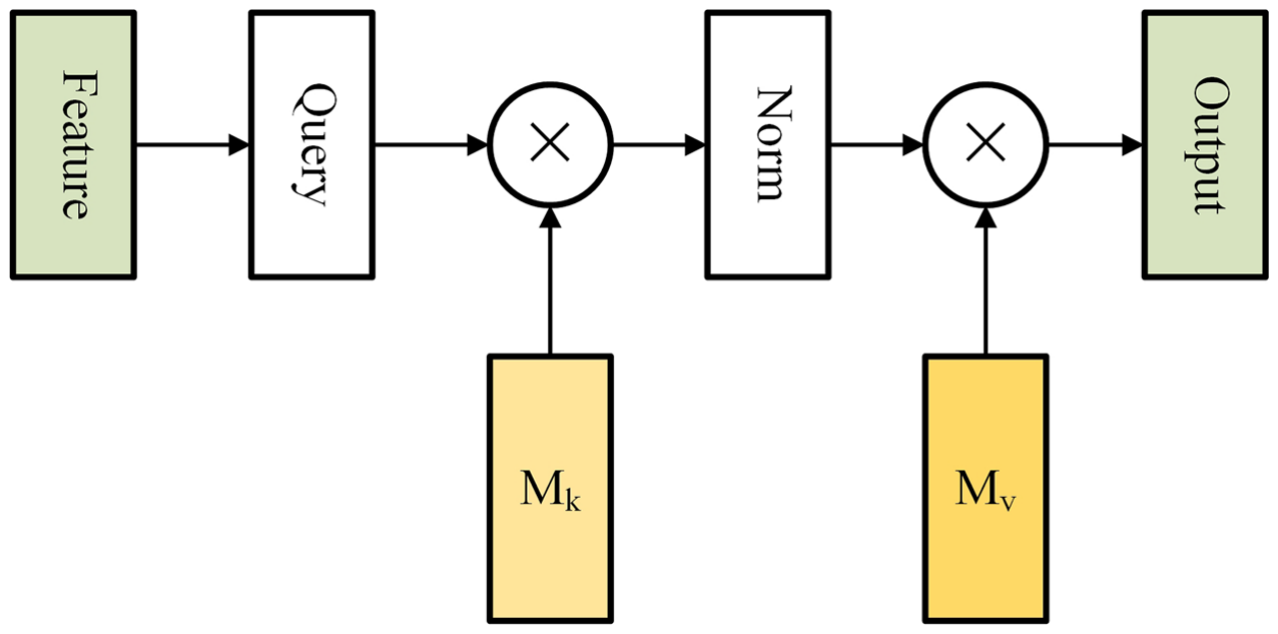
Figure shows the internal structure of external attention. The external attention mechanism calculates the correlation between input elements and assigns weights to each element. This allows the model to selectively focus on the most relevant elements. The mechanism is applicable to a wide range of tasks, including natural language processing, computer vision, and speech recognition.

Self-attention is an attention mechanism that captures long-range dependencies by calculating the correlation between all positions within a single sample (Vaswani et al., 2017). However, this method incurs high computational complexity and overlooks the inter-sample relationship. To tackle these challenges, Guo et al. proposed a new attention mechanism called external attention (EA). It is composed of solely two linear layers and two normalization layers, which optimize both accuracy and prediction time of our model (Figure 3).


$$A=\mathrm{Norm}\left(FM_{k}^{T}\right)$$

$$F_{\mathrm{out}}=AM_{v}$$

where $M_{k}$ and $M_{v}$ are learnable weight parameters. The relationship between different samples in the dataset can be established as each EMG window is generated under the same number and position of MU conditions. Therefore, it is crucial to include an attention mechanism in the model to establish the relationship between each window.

### 2.2 Data source and model building

#### 2.2.1 Simulated EMG signal

The volumetric conductor commonly used in studies is typically represented as a three-layer model with a cylinder shape, consisting of skin, fat, muscle, and other tissues (Farina and Merletti, 2001). These layers exhibit different conductive properties, with skin and fat being considered isotropic, while muscle tissue is considered anisotropic. Muscle tissue plays a significant role in the volume conductor. Alternatively, employing a single-layer muscle structure can accurately reproduce the motor unit action potential (MUAP) waveform and improve computational efficiency. As a result, the surface electromyography (EMG) signal generated in this study primarily acts as a volume conductor composed of single-layer muscle tissue.

It is assumed that MU locations are randomly distributed, and the muscle fibers innervated by each MU are also randomly distributed within a circular region. The muscle fiber density was set at 20 fibers per square millimeter (20/mm^2^), and the propagation speed of the MUAP was set at 4.0 m/s. The initial pulse rate of MU was 8 pulses per second (pps), and the peak pulse rate was 35pps. During the experiment, the muscle contraction force will be maintained at three fixed values: 10, 30, and 50% of the maximal voluntary contraction (MVC), and only Gaussian white noise was considered during data acquisition.

The signal collection instrument used in this study consisted of a high-density surface electrode array measuring 13 * 5. Each electrode for signal collection had a fixed radius of 1 mm, and the distance between any two adjacent electrodes was set at 5 mm. The data presented in this paper were obtained using the approach originally proposed by Fuglevand et al. (1993).

#### 2.2.2 Experimental EMG signal

We used the open-source data of Hug et al. (2020). The dataset includes EMG signals collected from the participant’s gastrocnemius muscle, as well as the decomposition results obtained from the gCKC algorithm. For the gastrocnemius muscle, the open-source data utilizes MATLAB files to record the decomposed EMG signals, binary Pulse Trains data of each successfully identified MU within the signal, force signals (not used in our experiment), sampling rate, IPT, pulse noise rates (PNRs) (Holobar et al., 2014), and other information. The Pulse Trains of each MU were manually edited and optimized. In the experiment, a total of 5 participants completed three types of contractions at 10, 30, and 50% of MVC. The data will be used as the data source for the experiment. The gastrocnemius medialis muscle was covered with a two-dimensional adhesive grid containing 64 electrodes (13 × 5 electrodes with one electrode absent in a corner, gold-coated, inter-electrode distance: 8 mm). EMG signals were recorded in unipolar mode, filtered with a bandpass ranging from 10 to 900 Hz, and digitized at a sampling rate of 2048 Hz.

#### 2.2.3 Data processing and model prediction method

**Figure 4:**
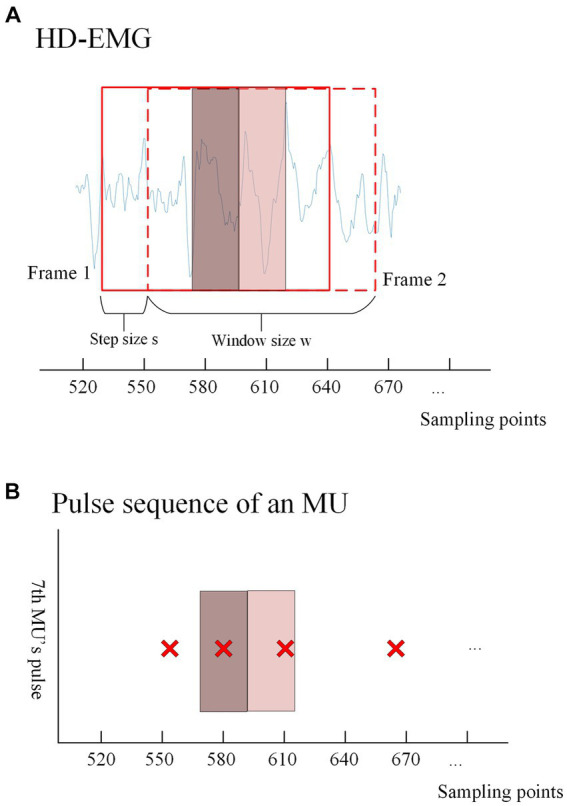
**(A)** Shows the windowed interception of HD-EMG signals for training data acquisition. An intercept with a length of ‘ $w^{’}$ is performed after every ‘ $s^{’}$ samples from a given sampling point, and each window overlaps. The solid box represents the first window (frame1) taken from the EMG, and the dashed box represents the second window (frame2) taken after shifting s samples. The dark red translucent box represents the range in which the first window can recognize pulse trains, and the light red translucent box represents the range in which the second window can recognize pulse. The discussion on the values of this range will be discussed in the Results section. The extraction method of training labels is shown in **(B)**, where the presence or absence of pulse issuance (0 or 1) of a particular MU within the center ‘ $s^{’}$ sampling points of each window is used as the training label according to the window interception method. **(B)** Shows the pulse trains obtained by the gCKC algorithm of the 7th MU. The red ‘x’s form the pulse trains of this MU, and each red ‘x’ represents a pulse recognized at this sampling point by the gCKC algorithm. The pulse inside the dark red transparent box indicates that the pulse can be recognized by the model in the frame1, and the pulse inside the light red transparent box indicates that the pulse can be recognized by the model in the frame2.

First, the gCKC algorithm is used to decompose the sEMG signals. Prior to decomposition, the signals are subjected to band-pass filtering and whitening, ranging from 10 Hz to 500 Hz. This decomposition yields pulse trains, which are essential for training the model. Next, the EMG signal is normalized to scale the data within a specified range. This normalization process helps accelerate the training process and improves the effectiveness of the model objectively. After normalization, the high-density EMG signal is divided into windows based on the sliding window principle (as shown in Figure 4). The window division is performed using the same channel number (which depends on the signal itself) and the same number of sampling points ‘w’ as the training data. The parameter ‘s’ in Figure 4 corresponds to the interval of samples at the beginning of each window. The optimal values for the aforementioned parameters will be analyzed and discussed in the results section.

In this experiment, the method used to identify pulses is by analyzing the sEMG information obtained from ‘w’ sampling points within the window. Specifically, the goal is to detect whether there is a pulse present at the ‘s’ sampling points located in the center of the window. For each MU, if a pulse is detected within the ‘s’ sampling points of the EMG signal, the label of the window is assigned as 1, indicating the presence of a pulse. Conversely, if no pulse is detected within those ‘s’ sampling points, the label is assigned as 0, indicating the absence of a pulse. To ensure that pulses are not missed within the ‘s’ sampling points, the size range of ‘s’ is carefully chosen, ranging from 5 to 40. The impact of different ‘s’ sizes will be discussed and analyzed in the results section.

The decomposition results of gCKC provide information about the state of each MU pulse within the specified time range of the window. According to the decomposition results of gCKC, if a pulse is detected within the sampling point range ‘s’ at the center of the window, its label is specified as 1, indicating the presence of a pulse. On the contrary, if no pulse is detected, it will be specified as 0, indicating no pulse.

#### 2.2.4 Model structure

For the provided training data and tags, we proposed two network structures: single-output GRU network (SO-GRU) and multi-output GRU network (MO-GRU).

SO-GRU predicts pulse trains for a particular MU (which can be specified prior to training), while MO-GRU can predict pulse trains for multiple MUs at the same time.

In summary, the main difference between SO-GRU and MO-GRU is that, in a task, it decomposes one or multiple MUs simultaneously.

**Figure 5:**
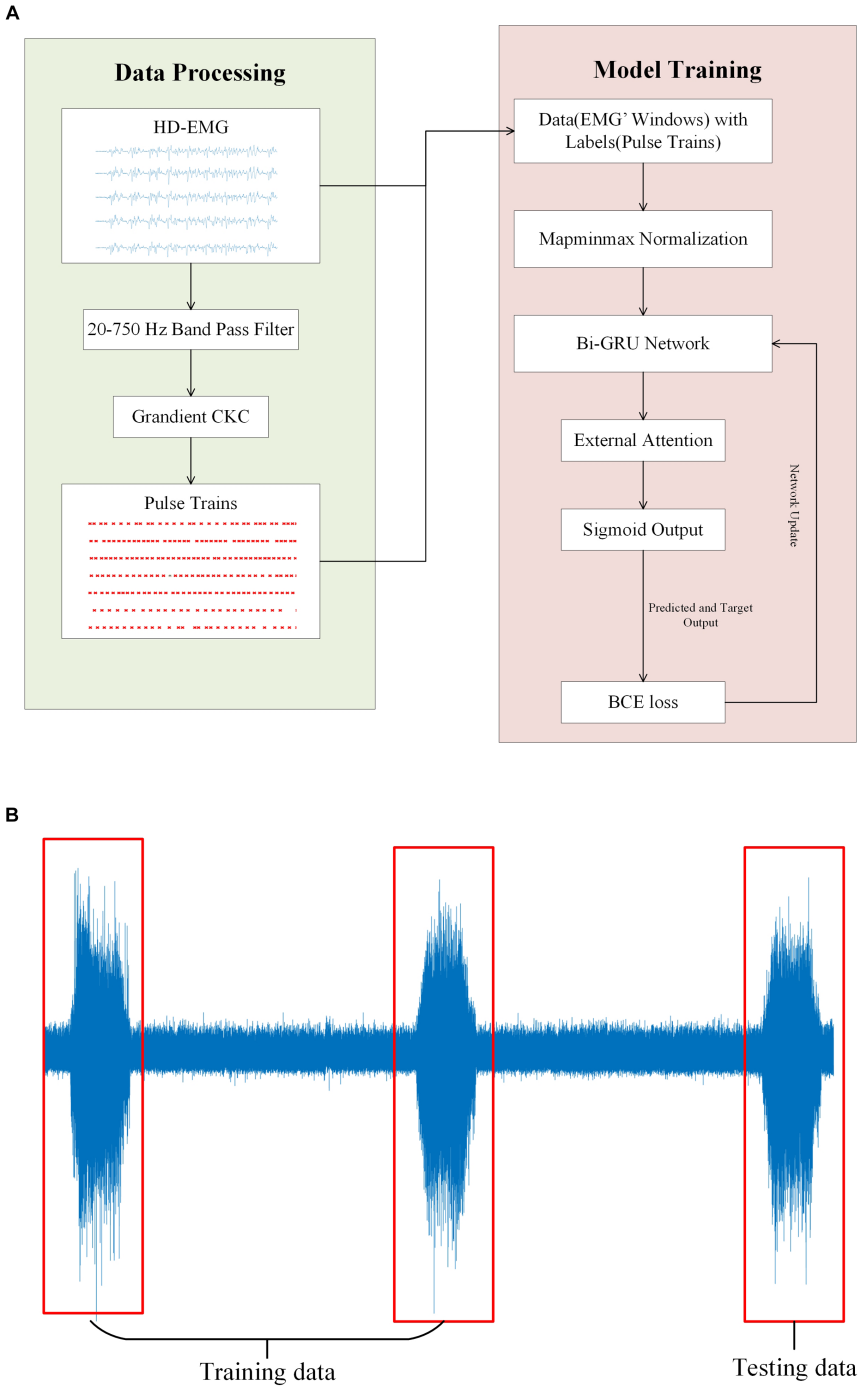
**(A)** Illustrates the experimental flow and the network model structure of this paper. The gCKC algorithm is used to decompose the HD-sEMG signal for the purpose of obtaining the pulse sequence. This pulse sequence along with the HD-sEMG signal is utilized as the data and label, respectively, during model training. **(B)** Depicts a participant executing three muscle contractions during a single signal acquisition. The training data was obtained from the initial two contractions of the EMG signals, with the final one being used as the test data.

For the model’s implementation, both structures depicted share numerous similarities (Figure 5A). Firstly, the input data comes in the form of a matrix of size w∗s . Upon passing through the GRU layer, the data transforms into a matrix of size w∗256 . Some parameters of the GRU layer: hidden layers 128, feature size 64, 2 layers. Secondly, the attention layer modifies the data content while retaining the original formatting. Additionally, subsequent processing through the full connection layer results in the data being transformed into a 128*1 matrix. After applying batch standardization and the full connection layer, the data is smoothed into an n∗1 matrix, where n represents the number of MU to be decomposed. This value may vary based on changes made by the user to the network structure. Finally, as the blind source separation problem addressed by this model is a multi-label classification problem, we utilize a sigmoid activation function for the output.

#### 2.2.5 Training and testing strategy

**Figure 6:**
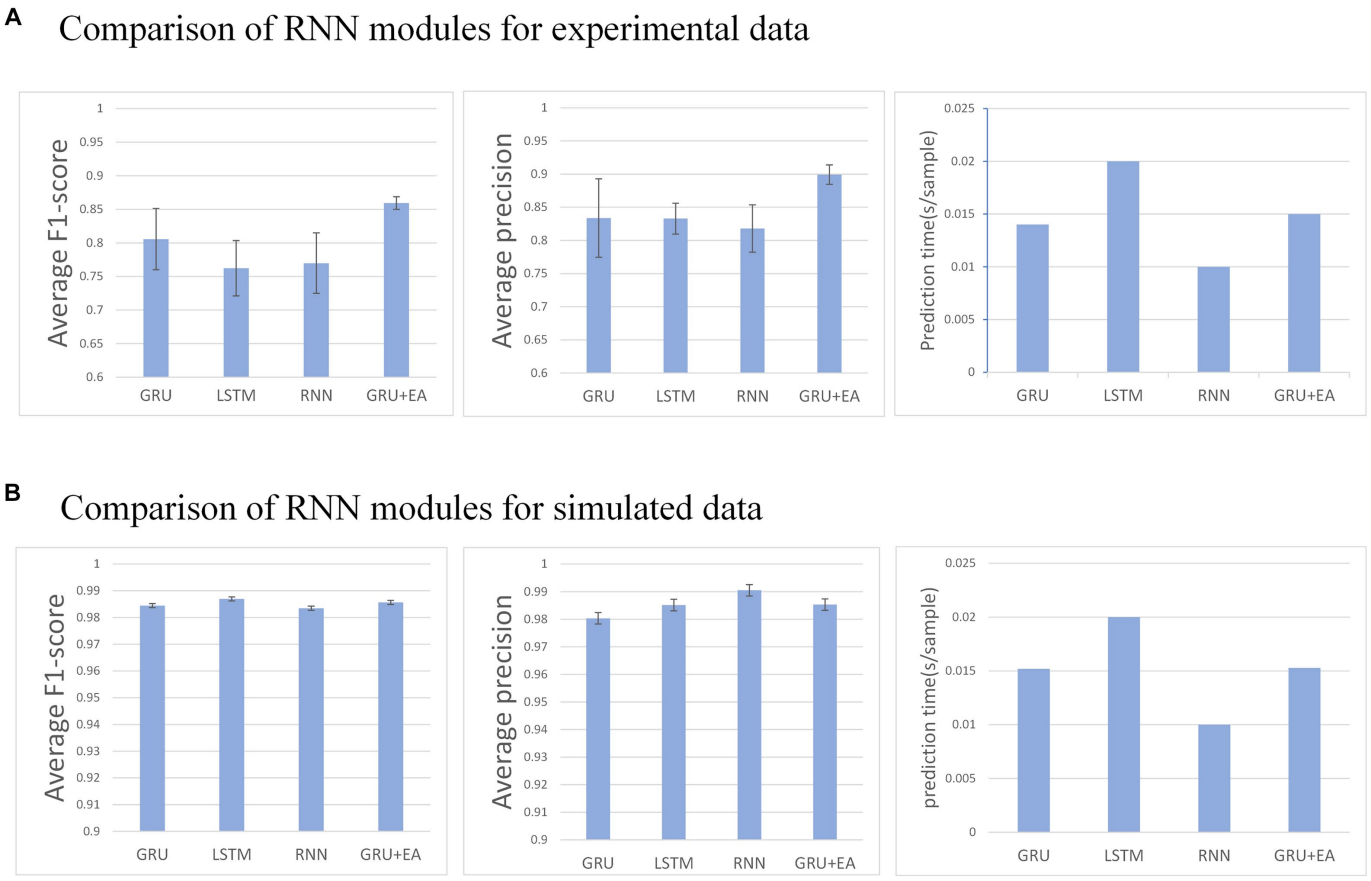
**(A)** Describes the performance of each recurrent neural module in the network on an experimental dataset, whereas **(B)** illustrates their performance on a simulated dataset. Any mu with an F1-score below 0.6 is disregarded.

On a personal computer with a CPU of AMD Radeon 6,800 h, a GPU of Nvidia RTX 3070Ti, and 16GB of memory, we utilized the Pytorch framework and its library to conduct model training. We categorized the entire EMG window and its corresponding tags into three groups, with the first two groups serving as the training datasets and the last group used for testing (Figure 5B). This step is critical to ensure that during the model testing process, certain data remains invisible to the model, leading to more accurate results. All following experiments will be conducted following this principle. During model training, the early stopping method is implemented. This entails terminating the training process and preserving the current model when the loss value of the test dataset either drops below a certain threshold or starts to increase (Figure 6).

For the trained model, we utilize two metrics to evaluate its performance: prediction accuracy and prediction time cost.

We employ two metrics to evaluate prediction accuracy: precision and F1-score:


$$\mathrm{Precision}=\frac{TP}{TP+FP}$$

$$\mathrm{Recall}=\frac{TP}{TP+FN}$$

$$F1-\mathrm{score}=\frac{2\mathrm{Precision}*\mathrm{Recall}}{\mathrm{Precision}+\mathrm{Recall}}$$

where TP is the number of windows that correctly predict the pulse, FP is the number of windows that are falsely predicted to have pulse, and FN is the number of windows that have pulse but are missed by the model. Precision and recall, respectively, measure the proportion of TP of the predicted pulse and the actual pulse. F1-score combines the two measures, which is more persuasive than accuracy.

As for predicting the time cost, we measure it by calculating the amount of time it takes to predict a sample. This process is executed using the relevant code in the integrated development environment.

In the following model training, we conducted ablation experiments on RNN and its variants modules, attention mechanism modules, MO and SO structures, SNR, and data processing methods.

## 3 Results

In the experimental data set, there were 5 participants in the experiment, and each participant provided the results of 10 MUs (a total of 50). While in the simulated data set, six MUs were decomposed each time, for a total of 5 times. The variance line of each experiment represents the degree of dispersion of the decomposition results of all MUs. For each MU, a F1-score above 0.6 will be accepted and those below will be disregarded unless further specified. The quantity of MUs and their PNRs, decomposed by the gCKC algorithm, together with their corresponding PNRs in both experimental and simulated data sets, are presented in Table 1. Due to the inability to obtain absolutely true and correct pulse trains from experimental data, we will uniformly use pulse trains obtained by the widely recognized gCKC algorithm as the gold standard for model decomposition performance evaluation. In the following experiment, we will use T-test for significance analysis, with the threshold set to 0.05.

**Table 1:** The table displays the decomposition of MUs for gCKC across the two datasets.

Data set	MU counts	PNR
Experimental data	50	37 ± 1
Simulated data	30	23 ± 1

Only MUs with a PNR greater than 20 will qualify for training the model.

### 3.1 Comparison of effects between recurrent neural networks

This section aims to evaluate the influence of different RNN modules on the predictive performance of the model. The RNN modules under investigation include GRU, LSTM, RNN, and GRU + EA. The experimental conditions for this evaluation are as follows: w=120 , step size of s=20 , and training in the MO-GRU. The SNR of the simulated data is 20.

The result indicates that all recurrent neural networks exhibit high accuracy (F1 score = 0.95 ± 0.01) in the simulated data set. However, in the experimental data set, GRU-external attention demonstrates the highest accuracy (average F1 score = 0.86) among all the networks. Conversely, LSTM performs inferiorly in this regard (F1 score = 0.76). In the experimental dataset, all networks except for LSTM were able to successfully decompose a good number of MUs (30–49 out of 50). Regarding prediction speed, RNN performed the fastest (0.01 s/sample), followed by GRU and its attention version (0.015 ± 0.001 s/sample), and the slowest was LSTM (0.02 s/sample). Analyzing the *p*-values, GRU + EA has a significant advantage.

### 3.2 Comparison of effects between attention mechanism

**Figure 7:**
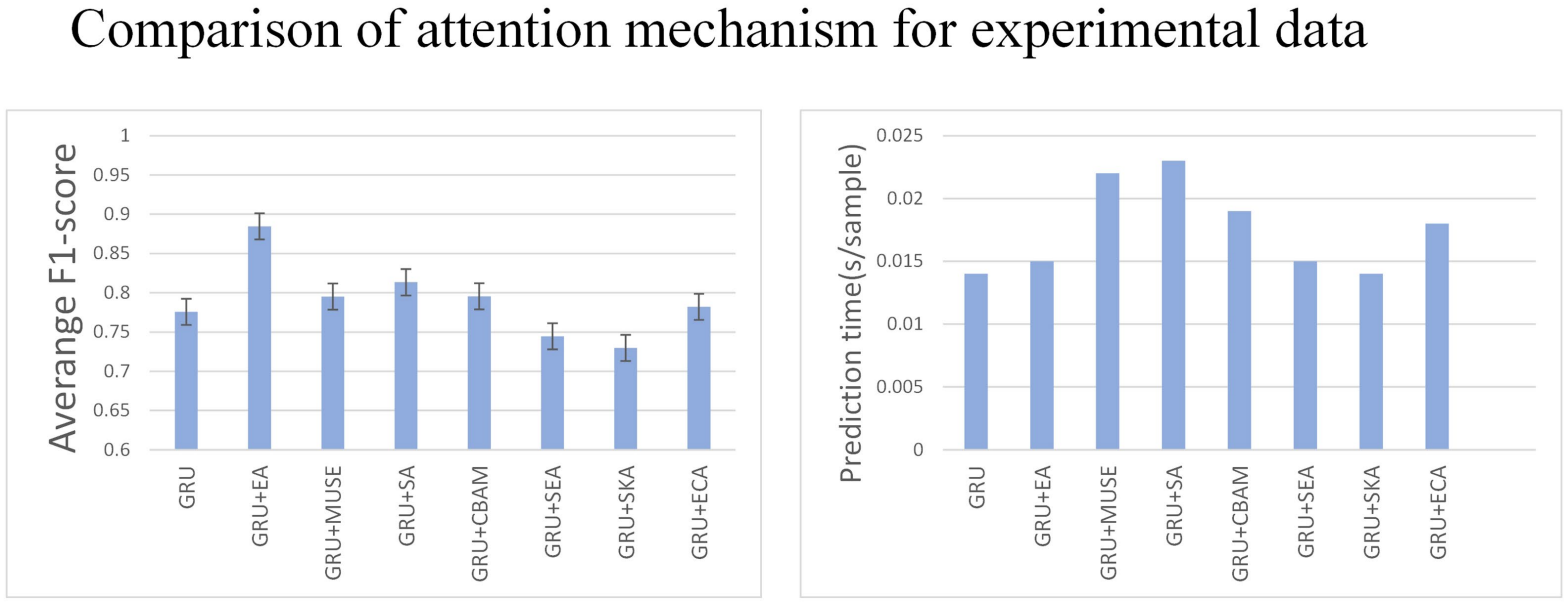
The figure shows the impact of each attention mechanism module on the network.

Various tests on recurrent neural networks showed that GRU achieved high accuracy and efficiency as shown in Figure 7. Subsequently, we examined how the addition of different types of attention affected GRU’s performance. We tested the effects of Effective Channel Attention (ECA) (Wang et al., 2020), Selective Kernel Attention (SKA) (Li et al., 2019), Squeeze and Excitation Attention (SEA) (Hu et al., 2018), Convolutional Block Attention Module (CBAM) (Woo et al., 2018), Self-Attention (SA) (Shaw et al., 2018), Multi-faceted attention-based Signed network Embedding framework (MUSE) (Yan et al., 2023), and External Attention (EA) (Bahdanau et al., 2014) on model prediction performance. The experiment was conducted on the MO-GRU model where the training data set had a window size of w=120 and a step size of s=20 . The SNR of the simulated data is 20.

Multiple combinations of attention and GRU were tested. External attention has the best advantage (average F1-score = 0.87, 0.015 s/sample), followed by CBAM attention (average F1-score = 0.78, 0.015 s/sample). The results indicate that External attention provided the greatest advantage with an F1-score of 0.87 and a sample prediction time of 0.015 s. However, the other attention decomposition effects were unsatisfactory, did not meet accuracy requirements, or incurred a long prediction time cost. Analyzing the *p*-values, GRU + EA has a significant advantage.

### 3.3 Comparison of effects between So-GRU and MO-GRU

**Figure 8:**
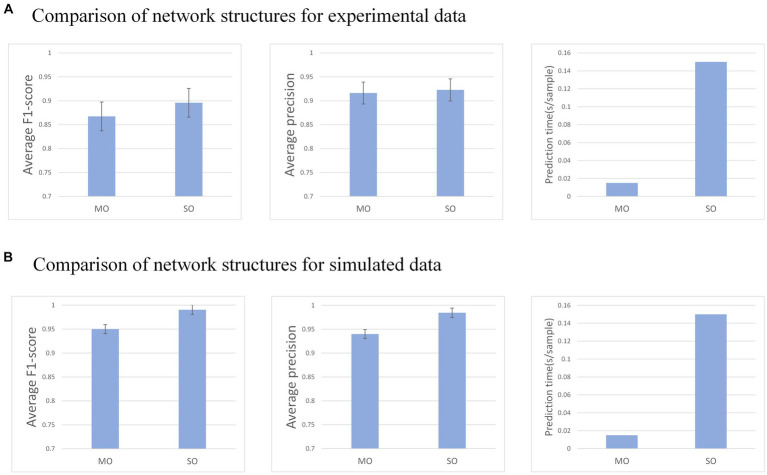
**(A)** and **(B)** show the MU decomposition of the real data set and the simulated data set in the MO and SO network structure.

After GRU external attention is the first choice in recurrent neural networks, we evaluated the performance of two network structures, SO-GRU and MO-GRU, as shown in Figure 8. This experiment will be conducted with simulation and experimental training data, utilizing a window size of w=120 and a step size of s=20 . The SNR of the simulated data is 20.

SO-GRU only outputs one MU’s decomposition result at a time, while MO-GRU can output five MU’s decomposition results simultaneously. Despite SO-GRU outperforming MO-GRU in both experimental data sets (average F1-score = 0.89 vs. 0.86) and simulated data sets (average F1-score = 0.86 vs. 0.98) concerning F1-score, its decomposition time costs are nearly five times those of MO-GRU (0.15 s vs. 0.03 s) when 10 MUs are decomposed. In the experimental data, analyzing the p-values, SO has a significant advantage in the F1-score.

### 3.4 Comparison of effects of signal noise rate

After choosing the external attention variant of MO-GRU, we introduce Gaussian white noise to the simulated data to evaluate the model’s robustness to noise. This simulation will be conducted with a simulated training data window size w=120 and the step size s=20 , and the six MU will be decomposed.

**Figure 9:**
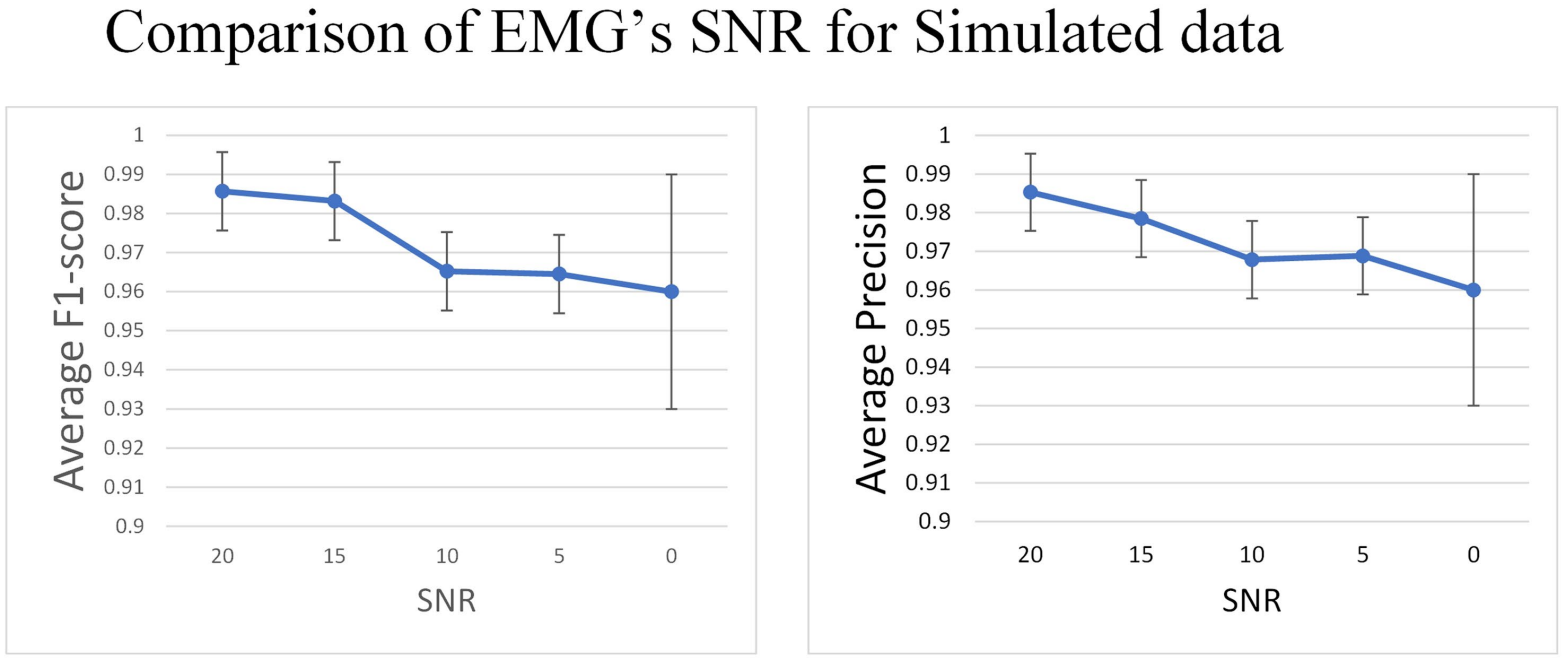
In the simulated data set, Gaussian white noise with a mean value of 0 is added to the signal to generate data with different SNR.

The experiment indicates that the average F1-score remains relatively stable (0.985 to 0.96) as the SNR decreases from 20 to 0 (Figure 9).

### 3.5 Comparison of effects of window size and step size

**Figure 10:**
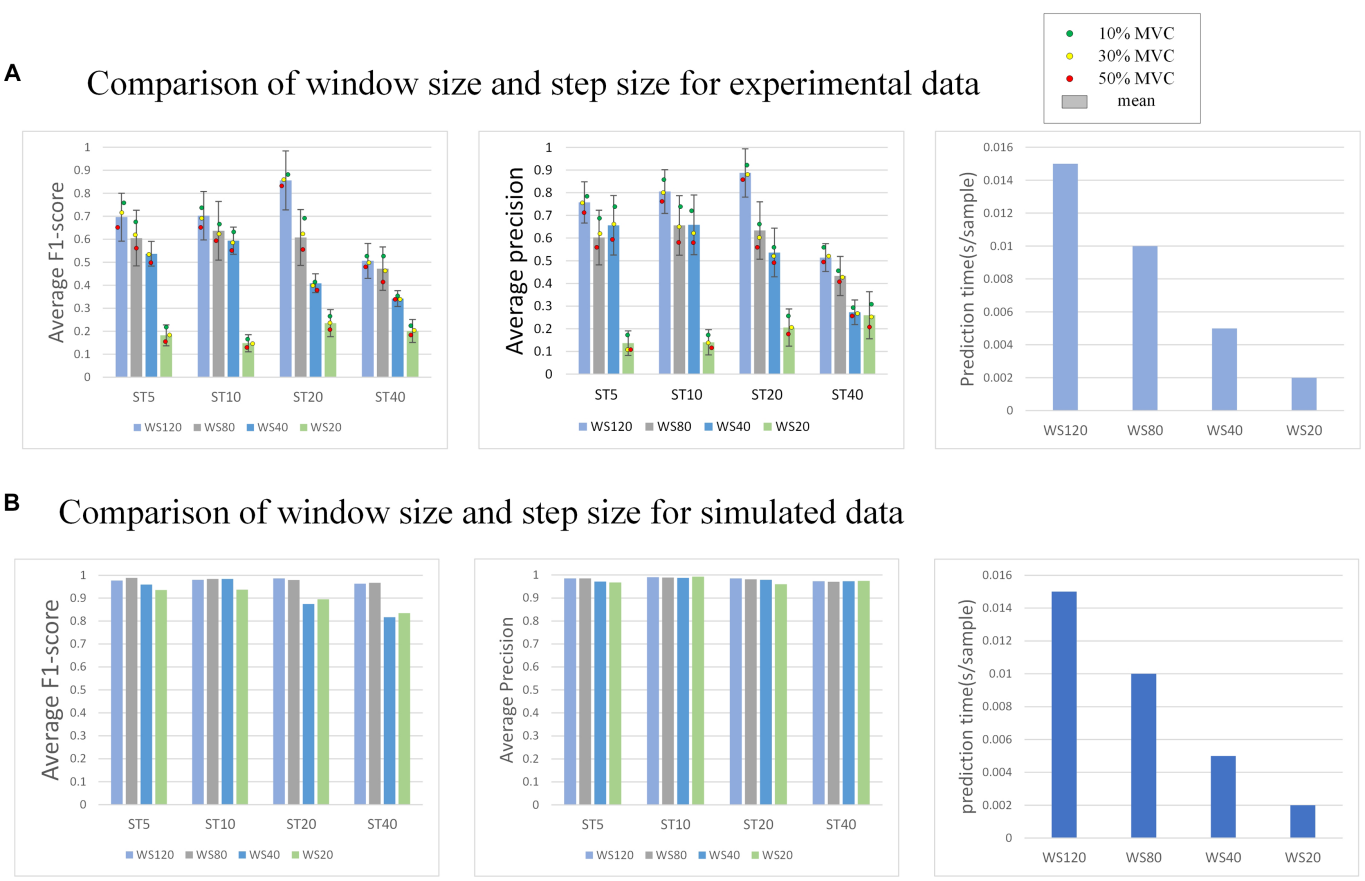
**(A)** Shows the performance of the model decomposition using varying window sizes and step sizes in the experimental dataset. **(B)** Shows the performance in the simulated dataset. The dots of different colors represent the mean values of all subjects at this MVC level. In **(B)**, the decomposition results of each MU are satisfactory, so no error annotation is performed.

In the external attention version of MO-GRU, various window sizes and step sizes are arranged and combined to determine the optimal combination for achieving decomposition effects (as shown in Figure 10). This section focuses on conducting experiments using both simulated data with a SNR of 20 and experimental data. Moreover, all decomposed MUs are included in the statistics, regardless of their successful decomposition (F1-score > 0.6).

The effectiveness and efficiency of MU decomposition show significant variability with changes in window and step size. Generally, a higher F1-score is observed with a step size of 20. Despite a longer prediction time per sample, the F1-score is highest with a window size of 120. As the window size decreases, the decomposition effect also worsens, indicating a reduction in the number of MU decomposed and accuracy. However, the prediction sample time becomes faster. As the step size increases, the decomposition effect initially improves, reaching its peak at 20, but the accuracy drops rapidly at 40.

## 4 Discussion

Thus far, all experiments have concluded, revealing several interesting results.

First, we compared the functionalities of GRU, LSTM, and RNN modules in the network and determined that GRU yielded the best results. Our discussion revealed that the fast prediction speed of RNN is counteracted by their lower accuracy, which can be attributed to their relatively simple network structure that requires fewer training parameters. Additionally, compared to the other two recurrent networks, there is currently no effective method to prevent gradient disappearance or explosion during model training in RNN. Therefore, RNN is not towel-suited for capturing long-term dependence, and the training parameters may struggle to fit the EMG signal training dataset well. Although LSTM models exhibit high accuracy, their prediction speed is slower than other models due to their more complex network structure. In contrast to RNN, LSTM models introduce gating mechanisms, such as input gate, forgetting gate, and output gate, which contribute to improve performance. Compared to RNN, GRU partially solve the gradient vanishing problem. However, they impose a more significant computational burden on training and prediction times. GRU is essentially a simpler variation of LSTM. By utilizing the update and reset gates, the LSTM’s gating mechanism becomes more streamlined, there by accelerating the convergence and prediction of the training model. Moreover, the accuracy is comparable to that of the LSTM, thereby making it ideal for our relatively small EMG training dataset, typically lasting for tens of seconds. Therefore, due to the GRU module’s high accuracy and quick decomposition speed, we choose to include it in our proposed network.

Subsequently, we conducted experiments to evaluate the impact of attention in our proposed network. We compared the performance of attention and non-attention models to illustrate the effectiveness of our proposed network. As detailed in the research background and related work section, external attention employs two one-dimensional convolutions to implement the memory unit, which aligns with the one-dimensional data output of our GRU unit. Additionally, this matches with the HD-EMG signal data set (two-dimensional data: number of channels * length). However, certain attention mechanisms with limited efficacy require two-dimensional convolution in their attention processes, whereas our EMG signal dataset employs a two-dimensional representation that necessitates dimensionality expansion operations, ultimately resulting in suboptimal experimental outcomes. Consequently, we incorporate external attention, which significantly enhances network accuracy with little effect on decomposition time.

Two network structures, SO-GRU and MO-GRU, were compared for their performance. SO-GRU showed higher average accuracy. This could be attributed to SO-GRU having only one MU decomposition task per model, where each model corresponds to MU one by one. Parameters in the model specialize in a single MU, thus improving the decomposition accuracy of each MU model. On the contrary, the MO-GRU model decomposes multiple MUs at once, resulting in lower decomposition accuracy due to the specialization of parameters for multiple units. For practical application, we suggest employing the SO-GRU network for precise identification of MU pulse if time is not a constraint. Conversely, for real-time prediction of multiple MUs, we advise using the MO-GRU network.

In the simulated dataset, the EMG signal with Gaussian noise added was deployed as both the training and test sets to evaluate the model’s capacity to accommodate noise. Results indicate a gradual decrease in F1-score as SNR decreases from 20 to 0, though the decrease range is insignificant (0.98 to 0.96). After discussing the issue, these findings suggest that the model has higher sensitivity in detecting the pulse directly from untreated EMG. Additionally, the model generates pulse trains instead of other regression values, resulting in minimal impact of noise on the model. Therefore, the model is somewhat robust to noise.

The model’s performance was tested using various window sizes ( w ) and step sizes ( s ), revealing that accuracy is highest when w and s are, respectively, 120 and 20. Pulse prediction requires additional sample points (w) around the focal points of s sample points in order to more precisely decompose MU. Thus, at the window size level, performance improves with larger w; this increases sample points the model can use, accuracy of pulse prediction within s sample points and the prediction time cost. Concerning step size level, a performance peak is reached when s is between 5 to 20, followed by a sharp decrease when s varies from 20 to 40. The reason for the lower accuracy rate may be due to the fact that when s is too small, even though the window moves with a small step and there is more data available, the prediction range s is also small, resulting in more stringent prediction conditions. Conversely, when s is too large, although the prediction conditions are relatively broad, the window moving step is too large, leading to less available training data. Due to the limited amount of data sets, the data has been underfitted resulting in accuracy not meeting the requirements. After a comprehensive consideration, it is recommended that the intermediate value s = 20 is an ideal option.

In summary, based on the test results, the recommended combination is w=120 and s=20 , taking into account both the accuracy and time cost of real-time task decomposition by MU. If time cost is not considered, specifically, real-time decomposition, we may increase the window size w to enhance accuracy. If you have varying amounts of training datasets, you may adjust the step size s accordingly to enhance the accuracy of decomposition or reduce the training time cost. Then, one advantage of this experiment is that using a small amount of data to train the model can achieve satisfactory accuracy. This experiment trained the model using EMG and its pulse trains with a collection time of 25 ± 10 s at a sampling rate of 2048 Hz. Finally, the network structure of the experiment in this article is relatively simple and easy to implement. We believe that the reasons behind the good performance in terms of accuracy and prediction speed of the model may stem from several factors: the ability of the GRU model to capture contextual relationships, the long-range dependency patterns captured by the EA mechanism, and the relatively simple network structure. Compared to the gCKC algorithm, which is considered the gold standard in the medical field, this model sacrifices a small amount of accuracy but significantly improves computational speed by eliminating the need for calculating the cross-correlation matrix and performing iterative computations. As a result, real-time decomposition of EMG signals becomes feasible. But due to the limitations of the model’s ability, this model does not have robustness for the MU decomposition task of dynamically changing MVC muscles. So, we still need to conduct research on this in our future work.

## Data availability statement

The original contributions presented in the study are included in the article/supplementary material, further inquiries can be directed to the corresponding author.

## Author contributions

CL: Conceptualization, Investigation, Resources, Software, Supervision, Writing – original draft, Writing – review & editing. CC: Data curation, Formal analysis, Methodology, Validation, Visualization, Writing – original draft, Writing – review & editing. ZC: Formal analysis, Investigation, Validation, Writing – review & editing. XZ: Formal analysis, Investigation, Validation, Writing – review & editing.
